# Local refinement mechanism for improved plant leaf segmentation in cluttered backgrounds

**DOI:** 10.3389/fpls.2023.1211075

**Published:** 2023-08-30

**Authors:** Ruihan Ma, Alvaro Fuentes, Sook Yoon, Woon Yong Lee, Sang Cheol Kim, Hyongsuk Kim, Dong Sun Park

**Affiliations:** ^1^ Department of Electronics Engineering, Jeonbuk National University, Jeonbuk, Republic of Korea; ^2^ Core Research Institute for Intelligent Robots, Jeonbuk National University, Jeonbuk, Republic of Korea; ^3^ Department of Computer Engineering, Mokpo National University, Jeonnam, Republic of Korea; ^4^ Department of Food Engineering Research, Intelligent Robot Studio Co. Ltd., Gyeonggi-do, Republic of Korea

**Keywords:** deep learning, leaf instance segmentation, cluttered background, filtering, plant phenotyping

## Abstract

Plant phenotyping is a critical field in agriculture, aiming to understand crop growth under specific conditions. Recent research uses images to describe plant characteristics by detecting visual information within organs such as leaves, flowers, stems, and fruits. However, processing data in real field conditions, with challenges such as image blurring and occlusion, requires improvement. This paper proposes a deep learning-based approach for leaf instance segmentation with a local refinement mechanism to enhance performance in cluttered backgrounds. The refinement mechanism employs Gaussian low-pass and High-boost filters to enhance target instances and can be applied to the training or testing dataset. An instance segmentation architecture generates segmented masks and detected areas, facilitating the derivation of phenotypic information, such as leaf count and size. Experimental results on a tomato leaf dataset demonstrate the system’s accuracy in segmenting target leaves despite complex backgrounds. The investigation of the refinement mechanism with different kernel sizes reveals that larger kernel sizes benefit the system’s ability to generate more leaf instances when using a High-boost filter, while prediction performance decays with larger Gaussian low-pass filter kernel sizes. This research addresses challenges in real greenhouse scenarios and enables automatic recognition of phenotypic data for smart agriculture. The proposed approach has the potential to enhance agricultural practices, ultimately leading to improved crop yields and productivity.

## 1 Introduction

Understanding the growth processes of plants is essential for optimizing crop cultivation conditions (Hilty et al., 2021). The interpretation of crop responses is often tied to environmental and nutritional factors, and visual observations of plant development play a significant role in this understanding (Heuvelink, 2005). These visual cues offer tangible evidence of a plant’s well-being and the effects of different conditions on its growth. However, comprehending the intricate processes involved in plant growth and development is not a trivial task. It demands a high level of expertise and intuition, acquired through experience and dedicated study. Researchers, agronomists, and farmers continually strive to deepen their knowledge of plant growth processes and develop innovative approaches to harness this understanding for sustainable and efficient agricultural practices (Costa et al., 2019).

Plant development processes, including stems, leaves, flowers, and fruit ripening, directly impact plant yield, quality, and quantity of final products. Phenotyping becomes indispensable in identifying these changes and understanding plant responses (Pieruschka and Schurr, 2019). For example, in tomato plants, critical phenotyping variables such as leaf color, shape, size, and stem diameter offer insights into the plant’s health, stress levels, and the potential presence of diseases or pests (Geelen et al., 2018).

Recent advances in computer vision and deep learning have prompted significant interest in plant-related research (Costa et al., 2019). Previous studies have successfully employed techniques (Liu and Wang, 2021) such as image classification, object detection, and instance segmentation for tasks such as detecting diseases and pests (Mohanty et al., 2016; Fuentes et al., 2017; Fuentes et al., 2018; Jiang et al., 2020; Fuentes et al., 2021; Dong et al., 2022), counting leaves (Farjon et al., 2021), and detecting fruits (Afonso et al., 2020). In relation to our research, Das et al. (2023) proposed an ensemble segmentation model with UNet as the base encoder–decoder for detecting coleoptile emergence time, showcasing its potential for phenotyping applications. Similarly, Yang et al. (2020) utilized the Mask Region-based Convolutional Neural Network (Mask R-CNN) architecture for leaf segmentation. The researchers conducted thorough investigations to identify optimal hyperparameters for both segmentation and classification techniques. Despite these significant achievements, the challenge of deploying systems in real-world scenarios with diverse variables and cluttered backgrounds persists (Barbedo, 2018).

In real-world scenarios, plant leaves often overlap or get occluded by other elements, making it challenging for segmentation models to accurately distinguish individual instances (Zhang and Zhang, 2023). Additionally, variations in lighting, shadows, and image quality, with issues like blurred leaves and noise in the images can impact the model’s ability to extract meaningful features for accurate segmentation (Rzanny et al., 2017). Moreover, the limited availability of annotated training data for specific plant species (Xu et al., 2023) and growth stages poses a significant challenge in achieving robust and generalized segmentation models (Okyere et al., 2023). Furthermore, existing methods may struggle with instances of varying sizes and shapes, leading to incomplete or inaccurate segmentation results (Yang et al., 2020). Addressing these problems is critical to advancing the field of plant leaf instance segmentation and enabling applications in precision agriculture and automated plant phenotyping.

**Figure 1:**
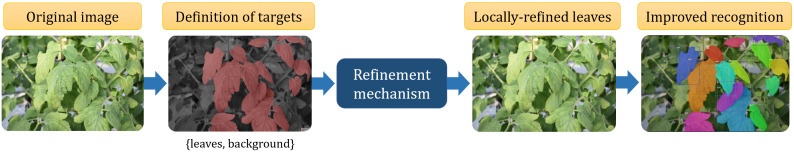
Overview of the proposed framework for instance segmentation of plant leaves in cluttered greenhouse backgrounds. It incorporates a refinement mechanism that operates locally on target areas, leading to enhanced recognition of individual leaf instances. The output results from this process allow us to derive essential phenotypic characteristics, including the accurate counting of leaves and the determination of their respective areas.

To address these technical gaps, this paper proposes a systematic deep learning-based approach for leaf instance segmentation in cluttered backgrounds. The study investigates the application of a filter-based instance refinement mechanism to enhance leaf instance segmentation, exploring its application on both training and testing data. **Figure 1** showcases the segmentation process of plant leaves within a cluttered greenhouse background. The proposed approach employs a refinement mechanism based that operates locally on target areas, leading to enhanced recognition of individual leaf instances. This refinement step is crucial for overcoming challenges related to occlusion, blurriness, and focus commonly encountered in real-world data collection scenarios. The output of the segmentation process provides segmented masks and bounding box information for each detected leaf instance. Leveraging these results, further processing is conducted to derive essential phenotypic characteristics, including the accurate counting of leaves and the determination of their respective areas. This comprehensive approach not only successfully identifies and segments plant leaves amidst cluttered backgrounds but also enables the extraction of critical phenotypic information that offers valuable insights into the plant’s health, growth, and overall performance. The results obtained from this figure demonstrate the effectiveness and potential of the proposed method for advancing plant phenotyping in greenhouse environments, contributing to the optimization of agricultural practices and crop management.

The contributions of this work are summarized as follows:

− A deep learning-based method for segmenting plant leaf instances, with instance segmentation and mask detection, is proposed and thoroughly validated on experiments conducted on our tomato plant dataset.
− The introduction of a simple yet effective local refinement mechanism based on filtering techniques applied locally to the leaf instances significantly improves the robustness of data used for training and testing, overcoming challenges related to data collection such as occlusion, blurriness, and focus.
− Our study offers a practical method for plant phenotyping using RGB images from real greenhouse environments, providing insights into data utilization for this application.

The rest of the paper is organized as follows: Related works on leaf instance segmentation and plant phenotyping techniques are reviewed in Section 2. The proposed method and strategy are introduced in Section 3. Experimental results, both qualitative and quantitative, are presented in Section 4. Finally, Section 5 concludes the research and outlines potential directions for future work.

## 2 Related works

This section presents an overview of the techniques used for leaf segmentation and plant phenotyping, including both traditional approaches and deep learning-based studies.

### 2.1 Traditional techniques for plant phenotyping

Plant phenotyping is a critical field in agriculture, providing valuable insights into crop growth and characteristics (Walter et al., 2015). Traditional methods have been utilized in this domain, including manual measurements of plant organ features and machine vision techniques for data collection (Kolhar and Jagtap, 2021). For instance, Praveen Kumar and Domnic (2019) employed statistical-based image enhancement, graph-based leaf region extraction, and circular Hough Transform for leaf counting. Zhang et al., 2018 explored plant segmentation using contour techniques and hand-crafted features, while Tian et al., 2019 used an adaptive K-means algorithm for tomato leaf image segmentation. Although these methods can be effective in controlled scenarios, their performance might be limited when applied in real-world situations with diverse variations and challenges.

As agriculture often involves cluttered backgrounds, occlusions, varying lighting conditions, and other complexities, these traditional approaches may struggle to handle the level of intricacy present in real-life environments. Consequently, the adoption of learnable approaches, such as deep learning, becomes more appropriate for tackling these challenging conditions (Xiong et al., 2021).

### 2.2 Leaf instance segmentation in cluttered backgrounds

In recent years, there has been a growing demand for systematic plant phenotyping, leading to increased interest in utilizing deep learning and computer vision-based techniques for image-based plant analysis (Costa et al., 2019; Fuentes et al., 2019). The main objective is to extract meaningful features from specific plant organs, such as leaves, flowers, stems, and fruits, to effectively characterize and evaluate their condition (Singh et al., 2018). Detection or segmentation architectures are commonly employed to provide detailed information at the instance level, such as bounding boxes (Dong et al., 2022) or masks (Xu et al., 2022), which prove valuable for applications such as plant disease and pest detection, as well as leaf, flower, or fruit counting.

The Leaf Segmentation Challenge (LSC) (Scharr et al., 2015) and the Workshop on Computer Vision Problems of Plant Phenotyping (CVPP) (Scharr et al., 2017) have significantly advanced plant phenotyping research. These initiatives aimed to develop state-of-the-art techniques for automatically obtaining phenotyping characteristics, with a particular focus on counting the number of leaves. As part of these efforts, they introduced new datasets with annotation labels for leaves and plants, inspiring various studies to address the challenge. For example, some researchers proposed methods for leaf segmentation using information like leaf borders, color, and texture features (Pape and Klukas, 2015), while others introduced neural network architectures for leaf counting (Aich and Stavness, 2017). Despite having limited training data, these approaches achieved satisfactory results. To tackle the issue of limited data availability, Kuznichov et al. (2019) explored data augmentation techniques to create synthetic samples based on existing data.

In the realm of plant segmentation with complex backgrounds, significant contributions have been made in recent years. For instance, Yang et al. (2020) employed Mask R-CNN with a VGG-16 feature extractor for leaf segmentation in complicated backgrounds, achieving a performance of 91.5%. The dataset used in their study consisted of images with clear leaf information, making leaves easily distinguishable from the background. Similarly, Br et al. (2021) proposed a segmentation method based on leaf images was proposed to identify the attributes of plant diseases. The researchers used a comprehensive dataset of various plant leaf images and developed a two-stream deep learning framework that accurately segments plants and counts leaves of different sizes and shapes. In Fan et al. (2022), the researchers introduced an auxiliary binary mask from the segmentation stream to enhance counting performance, reducing the impact of complex backgrounds. More recently, Lin et al. (2023) proposed a self-supervised semantic segmentation model that groups semantically similar pixels based on self-contained information, enabling a color-based leaf segmentation algorithm to identify leaf regions jointly. Furthermore, they introduced a self-supervised color correction model for images captured under complex illumination conditions.

While substantial progress has been made in plant leaf segmentation, most of the work has focused on outdoor environments, primarily due to the availability of datasets. In contrast, our research focuses on complex real-world greenhouse environments of tomato plants, where challenges such as leaf occlusions and varying scales are prevalent. To address these issues, we introduced a refinement mechanism based on filtering techniques, aiming to enhance the robustness of leaf instance segmentation and overcome the problem of image blurring. Our approach contributes to the advancement of plant phenotyping in challenging greenhouse settings and holds potential implications for agricultural practices and automation.

## 3 Proposed method

**Figure 2:**
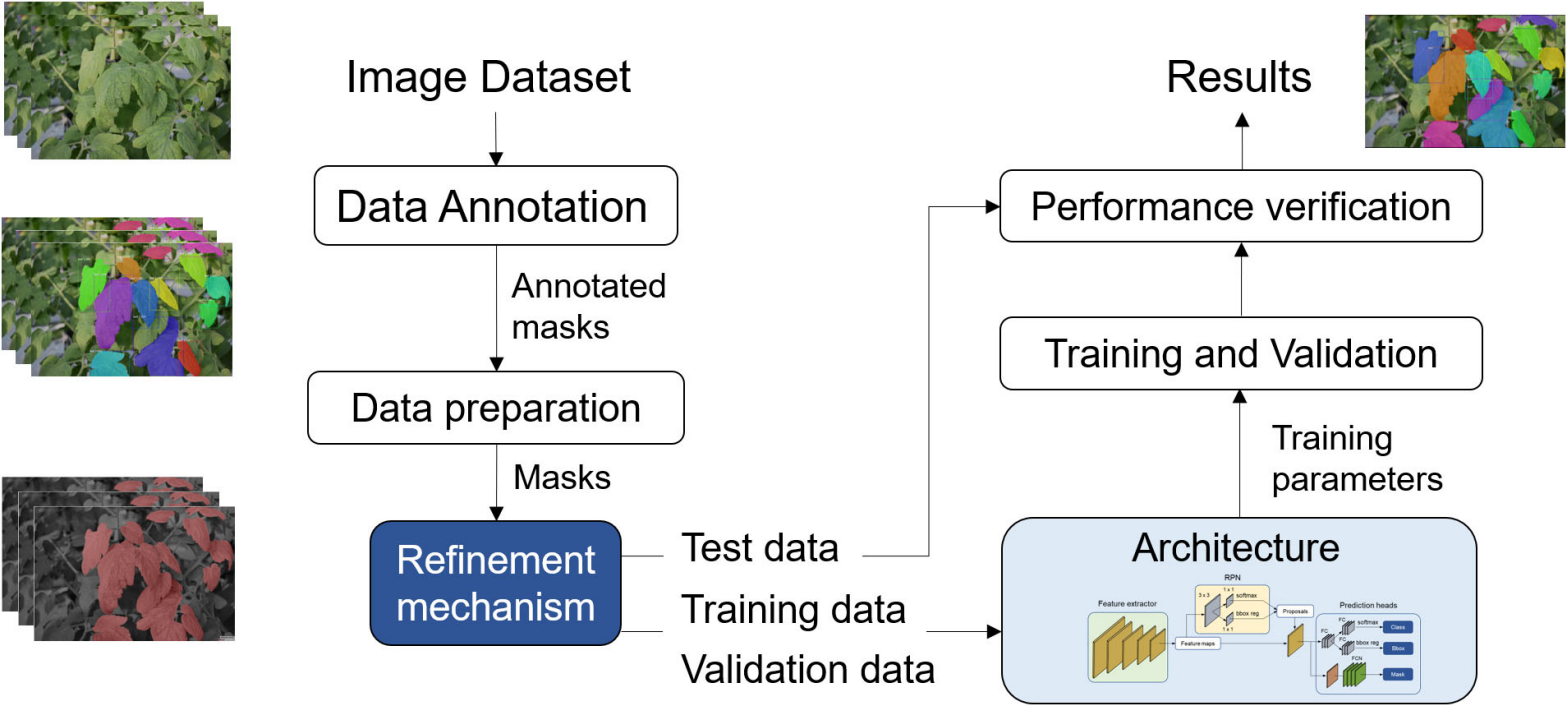
Overview of the proposed approach for plant leaf instance segmentation in cluttered backgrounds. The model encompasses two key elements: a refining mechanism directly applied to the data used for training or testing, and an instance segmentation architecture responsible for generating accurate leaf instances in the images.

This section provides a detailed explanation of the proposed approach and the techniques utilized for segmenting leaf instances in cluttered backgrounds. The primary architecture takes an input image and generates output results in the form of leaf instance masks. A pivotal aspect of our method is the data refinement mechanism, which enhances the robustness of the images used for both training and testing. This is achieved by locally applying filtering techniques to each target leaf instance. The implementation involves two distinct stages: one for training data and another for test data. An overview of the implementation process is illustrated in **Figure 2**.

### 3.1 Dataset description

**Figure 3:**
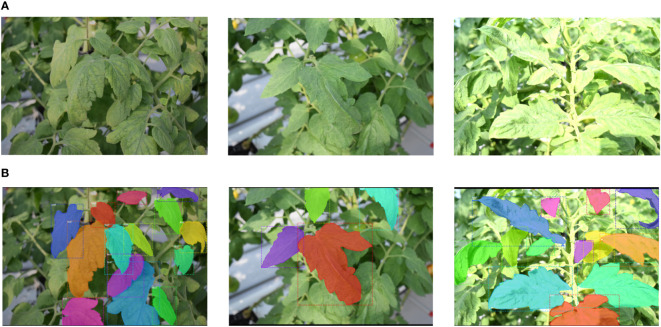
Examples of the tomato plants dataset, showcasing the images of the plants **(A)** alongside their corresponding mask annotations **(B)**. The mask annotations were applied to the foreground leaves, encompassing both clear and blurred samples, to provide comprehensive ground-truth data for the segmentation task.

In this study, we created a dataset specifically designed for the segmentation of leaf instances and the analysis of cluttered backgrounds. The dataset comprises 372 images of tomato plants, captured using multiple camera devices in various greenhouse environments. The images were taken under changing lighting conditions and feature diverse backgrounds. Each photo was captured parallel to the plants, encompassing surrounding areas as depicted in **Figure 3A**. The dataset exhibits complexities such as (1) variations in target leaf sizes and appearances, (2) different levels of leaf occlusion, and (3) blurred regions caused by camera movement and focus.

For generating ground-truth data, leaf regions were meticulously annotated using masks, regardless of their visual appearance, encompassing both well-defined and blurred samples. The annotations were performed manually utilizing an available toolbox for mask segmentation, as shown in **Figure 3B**. Overall, the annotations encompass 3,636 instances, with 2,045 instances allocated to the training set, 641 to the validation set, and 950 to the test set.

### 3.2 Instance segmentation architecture

**Figure 4:**
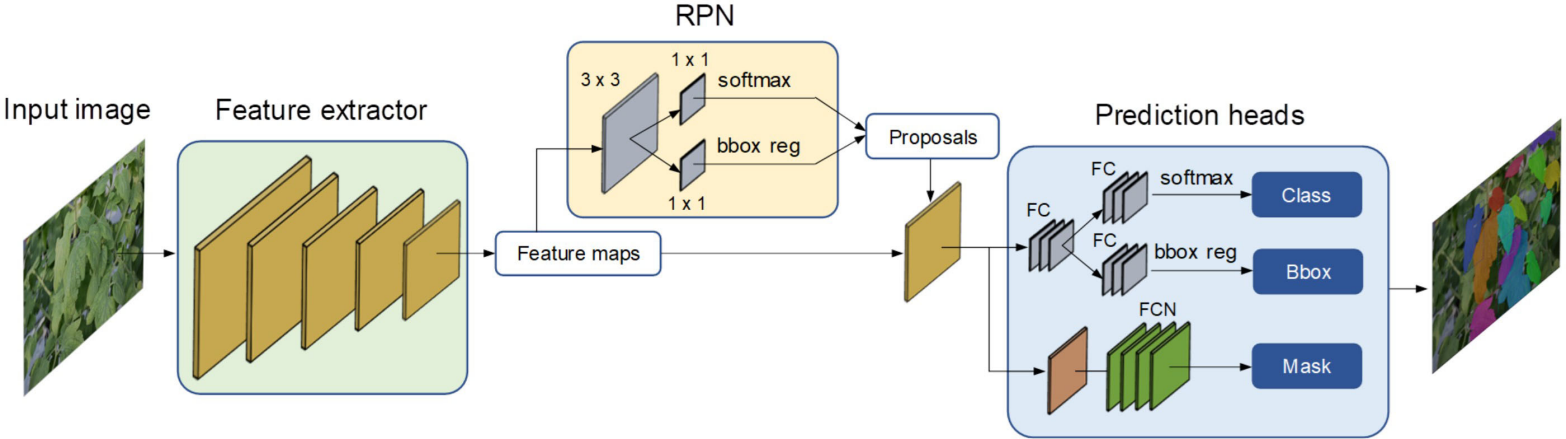
Instance segmentation architecture based on Mask R-CNN.

Leaf instance segmentation has been implemented using Mask R-CNN (He et al., 2017) as the core architecture. Mask R-CNN is a two-stage framework designed for both instance segmentation and object detection tasks. It leverages a Feature Pyramid Network (FPN) as its backbone to extract essential features from input images. In the first stage, a Region Proposal Network (RPN) generates Region of Interest (RoI) proposals, while in the second stage, Mask R-CNN predicts bounding boxes, class labels, and masks for each RoI. The overall architecture for leaf instance segmentation is illustrated in **Figure 4**.

During training, the end-to-end instance segmentation model aims to minimize the multi-task loss for each sampled RoI, which is composed of three components: classification loss ${\text{L}}_{\text{cls}}({\text{p}}_{\text{i}},{\text{p}}_{\text{i}}^{*})$ , bounding box regression ${\text{L}}_{\text{bbox}}(\text{t},{\text{t}}_{\text{i}}^{*})$ , and mask loss ***L_mask_*** as shown in Equation (1).


(1) $$\text{L}={\text{L}}_{\text{cls}}+{\text{L}}_{\text{bbo }}+{\text{L}}_{\text{mask}}$$

The classification loss is a logarithmic loss over two classes (object or not object) and is computed based on the output score ***p_i_*** of the classification branch for each anchor *i* and its corresponding ground-truth label ${\text{p}}_{\text{i}}^{*}$ .

The regression loss is activated only when the anchor contains an object. It computes the difference between the predicted bounding box parameters ***t_i_*** and the ground-truth parameters ${\text{t}}_{\text{i}}^{*}$ , which include four variables **[*t_x_*,*t_y_*,*t_w_*,*t_h_***], where **(*x*,*y*)** are the coordinates of the bounding box center, and its width and height **(*w*,*h*)**.

The mask loss is an average binary cross-entropy loss applied to the dedicated mask branch. As an instance segmentation approach, ***L_mask_*** utilizes the classification branch to allow the network to generate masks for each class separately, avoiding confusion among different categories.

### 3.3 Proposed local refinement mechanism

During data collection for our application, camera focus and blur were the most common image quality issues. These issues had a significant impact, particularly when dealing with cluttered background conditions and defining target areas accurately. Our research aims to address this challenge by introducing a “local refinement mechanism,” a simple yet effective technique that enhances the robustness of training and test data. The goal is to enable the system to accurately segment leaves regardless of background information.

After obtaining the annotated dataset, we applied the local refinement mechanism to the instances in both the training and test data. The main methods involved using Gaussian low-pass filtering and High-boost filtering, either independently or in combination, to improve the system’s recognition capabilities.

#### 3.3.1 Gaussian low-pass filter

GLPF allows transmitting signals with lower frequency, thereby helping to reduce noise and blurring regions in the image (Gonzalez and Woods, 2018). It smooths the image by averaging nearby pixels within a local region, reducing the disparity between pixel values. The effect of image blurring results is larger, as the smoothing mask also becomes larger. The GLPF generates blurring instance regions to assess the model’s ability to segment leaves under these conditions. Equation (2) specifies a GLPF:


(2) $$f_{G}(x,y)=\frac{1}{2\pi \sigma^{2}}e^{-\frac{x^{2}+y^{2}}{2\sigma^{2}}}$$

where *x* is the distance from the center on the horizontal axis, *y* is the distance from the center to the vertical axis, and σ is the standard deviation of the Gaussian distribution.

#### 3.3.2 High-boost filter

HBF emphasizes high-frequency image details without eliminating low-frequency components. It sharpens the image and enhances edges (Gonzalez and Woods, 2018). Multiplying the original image by an amplification factor *A* yields the definition of an HBF. The value of *A* determines the nature of the HBF, where higher values lead to brighter backgrounds, resulting in noise enhancement and image sharpening. Equation (3) defines the HBF:


(3) $$f_{HB}(x,y)=(A-1)f(x,y)+f_{hp}(x,y)$$

where *A* represents the amplification factor, and *f_hp_* is a high-pass filter. We applied the HBF locally to leaf instances to improve their regions’ sharpness, facilitating leaf boundary detection, especially in cases with occlusion. We experimented with different kernel sizes to find the optimal value for our approach.

**Figure 5:**
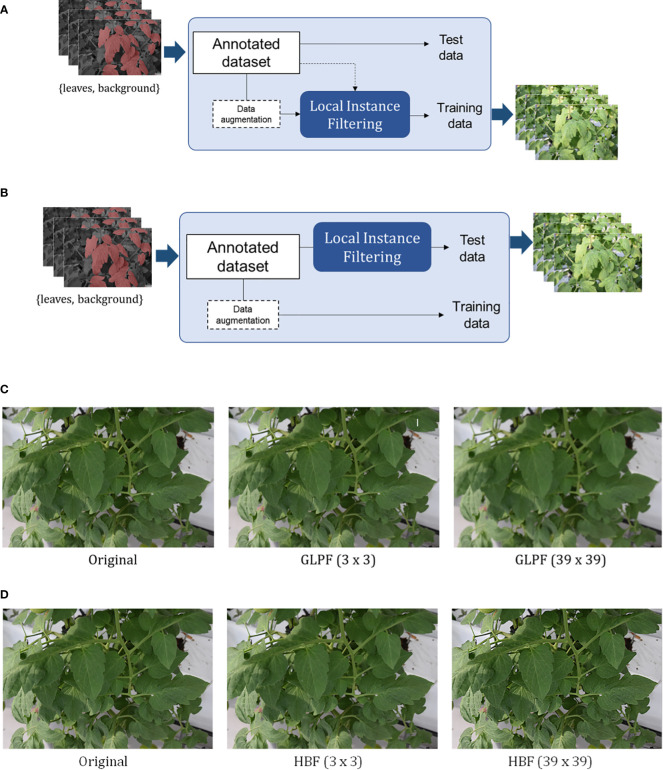
Application of the local refinement mechanism either on the training dataset **(A)** or the test dataset **(B)**. The impact of the filters with different kernel sizes on the images is demonstrated in the examples presented in **(C, D)** for the Gaussian low-pass and High-boost filters, respectively. [See **Figures A1** and **A2** in the Appendix for more detailed representations of the schemes in **(A, B)**].

We devised two scenarios for applying the refinement mechanism:

− Scenario 1: We aimed to determine whether applying the refinement mechanism enhances the robustness of features in the training dataset, as shown in **Figure 5B**.
− Scenario 2: We applied the refinement mechanism to the test data to assess whether the features from the training dataset effectively handle changes in the test data, as shown in **Figure 5B**.

**Figure A1:**
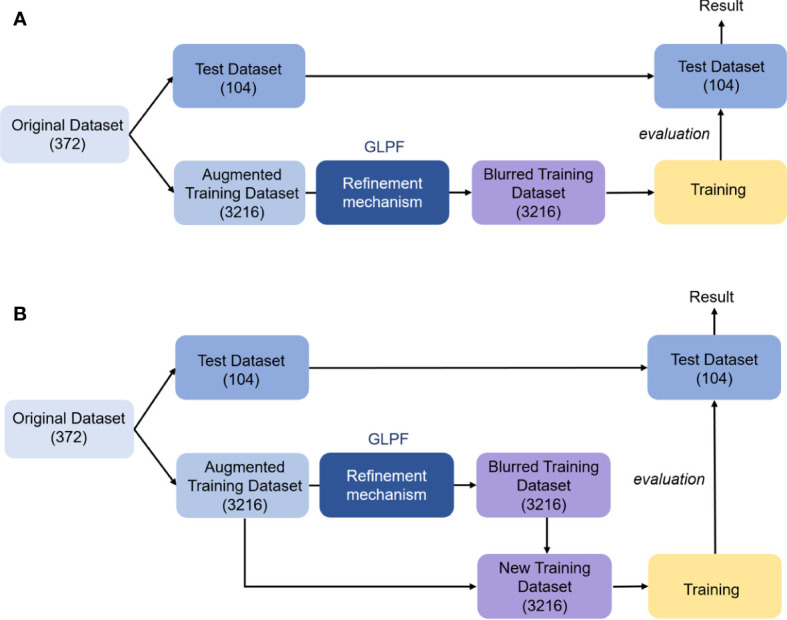
Local refinement mechanism (GLPF) applied to the augmented training dataset. **(A)** Applying the mechanism to the original images. **(B)** Combining the blurred dataset with the original images. The number inside the parenthesis shows the number of images.

**Figure A2:**
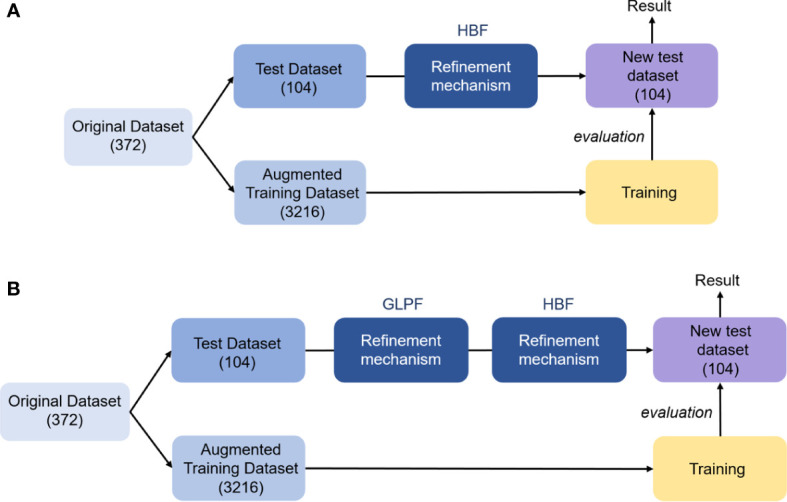
Local refinement mechanism applied to the test dataset. **(A)** HBF. **(B)** GLPF followed by HBF. The number inside the parenthesis shows the number of images.

We evaluated the system’s response to these changes by applying the local refinement filter with different kernel sizes. **Figures 5C, D** illustrate example images after applying the GLPF and HBF, respectively. In Section 4, we present the qualitative and quantitative results of our approach. Additional specific illustrations of the applied local refinement mechanism can be found in **Figures A1** and **A2** of the Appendix. These figures showcase how the mechanism is implemented on both the training and test datasets.

To avoid overfitting, data augmentation techniques were employed to increase the number of images in the training dataset on the two aforementioned cases. From this point onwards, we will use the abbreviation (ATD) to refer to the augmented training dataset. We used both online and offline data augmentation, including intensity and geometric transformations. Specifically, online data augmentation was executed during training, applying operations such as horizontal flip, Gaussian blur, brightness and contrast enhancement, and pixel loss. Offline data augmentation, performed as a separate process to the entire dataset before training, generated more images using techniques such as brightness and contrast enhancement, pixel dropout, horizontal flipping, rotation, and random combinations of all of them.

### 3.4 Evaluation metrics

We evaluated the performance of the proposed model using the Intersection Over Union (IoU) thresholding operation and the mean Average Precision (mAP) metric (Everingham et al., 2009). The standard MS COCO metrics were used for instance segmentation and bounding box detection. The mAP is calculated by computing the AP for each class and then averaging across all classes, taking into account the trade-off between precision and recall, and considering false positives (FPs) and false negatives (FNs). Equation (4) presents the formula for the mAP calculation.


(4) $$\text{mAP}=\frac{1}{\text{N}}\sum_{\text{i}=1}^{\text{N}}\text{A}{\text{P}}_{\text{i}}$$

Our primary focus in this evaluation was on the system’s ability to accurately identify leaf instances and potentially predict more leaf samples than those available in the training dataset. We present the results of our experiments in the following section to support our claims.

## 4 Experimental results

In this section, we provide the implementation details and present both quantitative and qualitative experimental results on the tomato plants dataset. These evaluations demonstrate the performance of our applied strategy in real-field scenarios.

### 4.1 Implementation details

For our implementation, we fine-tuned the model end to end using a pre-trained model on the MS-COCO dataset. To train the network, we utilized Stochastic Gradient Descent (SGD) along with the Adam optimizer, setting the learning rate to 0.000125, momentum to 0.9, and weight decay to 1e-4. After training the model for 50 epochs, we obtained the final instance segmentation weights. The training process was conducted on a computer equipped with 4 GPUs Titan RTX.

**Figure A3:**
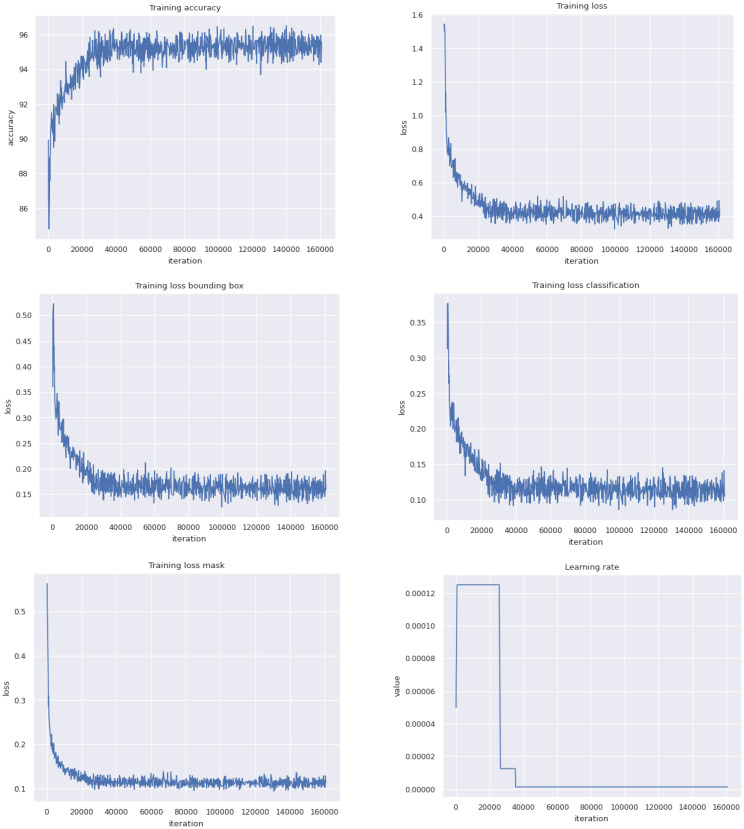
Training curves of the model.

The original images had a size of (4,032 and 3,024), and we resized the input images to (1,333 and 1,000). For implementation, we used the PyTorch framework, where the input tensor size was (6, 3, 1,333, and 1,000), which corresponds to the batch size, number of channels, width, and height, respectively. The first layer of the network used a 7 × 7 kernel size with a stride of 2. In the following convolutional layers, the kernel size was predominantly 3 × 3, and the stride was either 1 or 2, depending on the layer. In the Feature Pyramid Newtok (FPN), 1 × 1 and 3 × 3 convolutional layers were used. ReLU was applied after each convolutional layer to introduce non-linearity into the model. In the final stage of Mask R-CNN, a sigmoid activation function was used in the mask branch. The training curves of the model are presented in **Figure A3** in the Appendix.

### 4.2 Backbone feature extractor

We initiated our experiments by comparing the performance of different backbone architectures, namely, ResNet-18, ResNet-34, ResNet-50, and ResNet-101, to determine the most suitable one for our specific application. For this comparison, we directly trained the model using the original images without applying the local refinement mechanism on the leaf instances. The results of this evaluation are presented in **Table 1**. Among the tested networks, ResNet-50 demonstrated the highest performance in segmenting instance leaves, achieving an IoU > 0.5 of 91.6%. Our findings indicated that Mask R-CNN benefited significantly from deeper networks, particularly ResNet-50. As a result, we selected this architecture as the baseline backbone to conduct further experiments.

**Table 1:** Backbone architecture.

Model	Segmentation			Bounding box detection		
	AP_50_	AP_75_	AP_50–95_	AP_50_	AP_75_	AP_50–95_
ResNet-18	73.0	37.2	38.6	72.6	29.9	35.6
ResNet-34	82.1	51.6	48.1	81.4	48.8	46.7
ResNet-50	91.6	83.4	74.5	91.3	81.4	71.6
ResNet-101	90.2	75.6	67.2	89.0	69.2	60.9

### 4.3 Refinement mechanism applied to the training dataset

In this experiment, we focused on evaluating the first scenario presented in Section 3.3 and illustrated in **Figure 5A**. The goal was to assess the impact of the refinement mechanism when applied to the local leaf instances of the training dataset, with the intention of emulating the presence of blurry leaves in the data. By introducing blurriness, we aimed to generate the necessary features that would allow the model to perform well on the original test dataset, which contains instances of leaves with clearer visual appearance.

To achieve this, we utilized a GLPF in two different configurations:

#### 4.3.1 Refinement mechanism applied to the augmented training dataset

In this configuration, the model was trained on the augmented training dataset, which included instances of leaves with varying levels of blurriness introduced through the GLPF. The objective here was to assess the model’s ability to generalize effectively on the test data, which comprises images of original uncorrupted leaves. **Figure A1 A** in the Appendix illustrates the implemented strategy for this scenario.

#### 4.3.2 Refinement mechanism applied to the augmented training dataset and combined with the original samples

In this case, we combined the blurred dataset with the original augmented dataset. The purpose was to provide the model with more detailed features of the target areas, and the refinement mechanism acted as a type of data augmentation technique. However, for our specific task, we aimed to examine its impact as part of a partially corrupted dataset. **Figure A1 B** in the Appendix shows the strategy implemented for this configuration.

To comprehensively evaluate the model’s performance under different settings, we conducted a thorough analysis involving the number of predicted masks corresponding to leaves and the AP on the test dataset. This evaluation was carried out by applying various kernel sizes for the GLPF, which introduced multiple levels of blurring in the training data. To ensure the reliability of our findings, we conducted three rounds of model training and calculated the standard deviation.

The results presented in **Table 2** unveiled two prominent trends: In the first scenario, where the refinement mechanism was applied solely to the ATD, we observed a slight reduction in AP. However, an interesting phenomenon occurred; the model seemed to learn to associate the noise generated by applying the GLPF. Consequently, while the AP decreased slightly, the number of detected masks increased. This intriguing observation suggests that the model acquired enhanced capabilities to handle such blurred data during training, thereby becoming more robust against such changes.

**Table 2:** Results of the refinement mechanism applied to the training dataset.

Model	Kernel size	Predicted masks	Segmentation			Bounding box detection		
			AP_50_	AP_75_	AP_50–95_	AP_50_	AP_75_	AP_50–95_
Baseline	–	889 ± 11	91.5 ± 0.4	83.5 ± 0.5	74.5 ± 0.1	91.2 ± 0.3	80.9 ± 1.4	71.4 ± 0.7
GLPF on applied to the ATD	5	897 ± 3	91.9 ± 0.1	83.3 ± 1.8	75.0 ± 0.3	91.3 ± 0.8	80.4 ± 1.1	71.4 ± 0.3
	7	909 ± 4	91.3 ± 0.2	83.3 ± 1.0	74.6 ± 0.3	91.2 ± 0.2	80.5 ± 0.3	71.1 ± 0.4
	9	915 ± 4	91.5 ± 0.1	83.3 ± 0.5	74.6 ± 0.2	91.1 ± 0.2	80.3 ± 1.2	71.1 ± 0.1
GLPF applied to the ATD + OI	5	863 ± 4	89.7 ± 0.4	82.2 ± 0.8	74.4 ± 0.1	88.9 ± 0.6	79.3 ± 1.0	0.9 ± 0.2
	7	865 ± 7	89.7 ± 0.3	82.2 ± 1.1	74.2 ± 0.2	89.4 ± 0.2	79.2 ± 0.6	70.8 ± 0.1
	9	870 ± 2	90.1 ± 0.8	82.2 ± 1.0	74.3 ± 0.6	89.2 ± 0.3	79.6 ± 0.2	71.2 ± 0.9

ATD, Augmented training dataset.

OI, Original images.

In contrast, the second scenario, where original data were combined with the ATD, revealed a different outcome. Here, the performance of the model decreased, accompanied by a decline in the number of predicted masks. This decline can be attributed to the model’s primary focus on recognizing clear data. Consequently, when confronted with blurred data, the model became frequently confused, leading to a drop in performance. As the kernel size for the GLPF increased and blurring became more severe, this confusion further exacerbated the model’s inability to accurately segment leaves.

**Figure 6:**
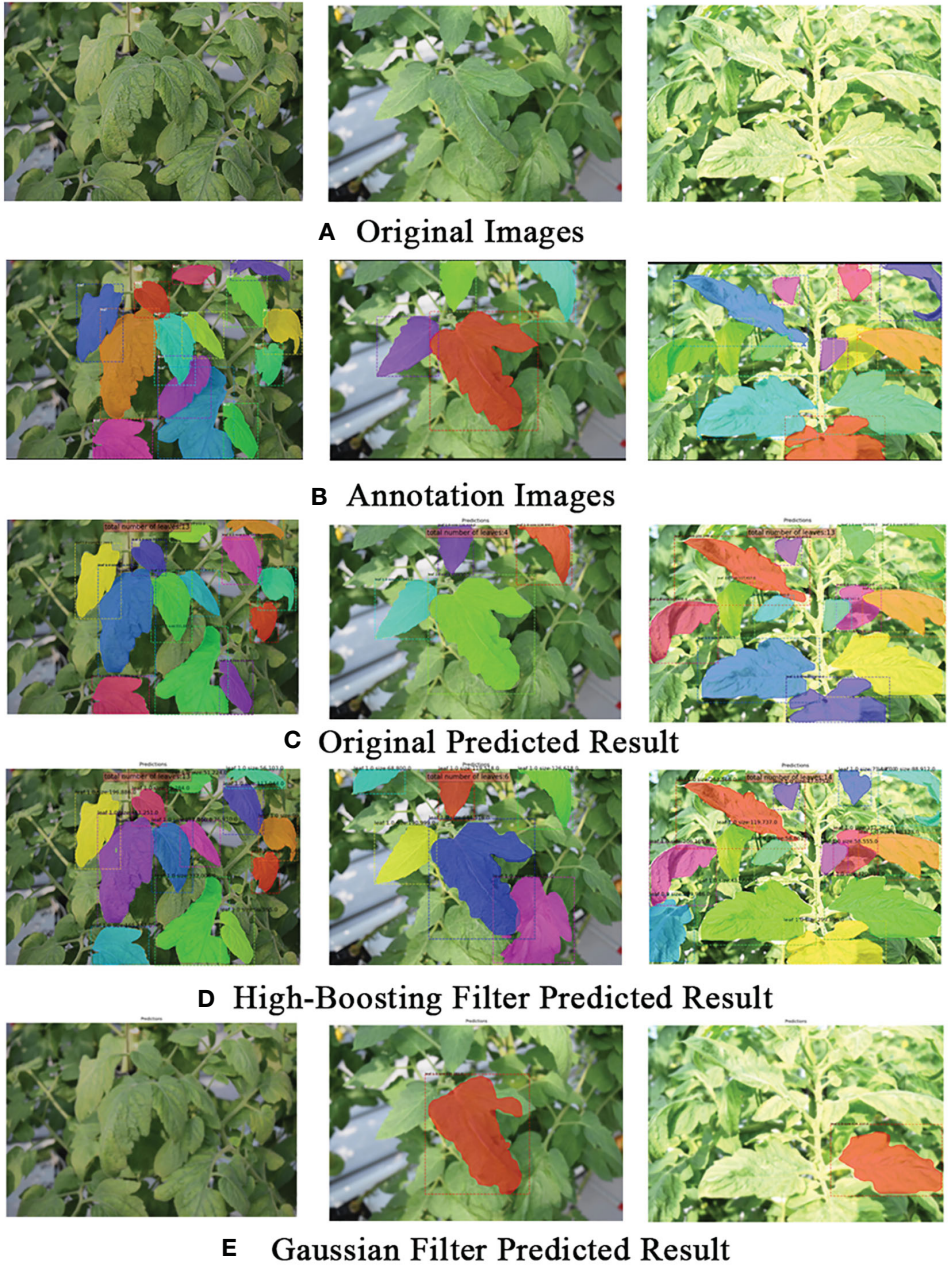
Example qualitative results on the tomato plant dataset. **(A)** Original images. **(B)** Ground truth (actual annotations). **(C)** Predicted results on the original images. **(D)** Predicted results using Gaussian low-pass filter on the training dataset. **(E)** Predicted results using the High-boost filter on the test dataset. The visual comparison highlights how different approaches, such as applying filters to the training or test datasets, influence the model’s predictions.

These findings strongly indicate that the blurring data introduced by the GLPF, when applied to the training dataset, significantly contributed to making the model robust against blurring effects in the data. Consequently, this adaptation played a vital role in improving the model’s ability to accurately segment leaves. **Figure 6** provided some qualitative examples of the model’s performance, further highlighting the challenges and limitations posed by introducing blurriness in the training dataset.

This study showcased the significance of the refinement mechanism, particularly when applied to the ATD, in enhancing the model’s robustness against blurriness in the data, leading to improved leaf instance segmentation performance. However, caution is required when combining original and blurred data during training, as it may adversely affect the model’s ability to handle blurriness. These insights have practical implications for real-world applications.

### 4.4 Refinement mechanism applied to the test data

In the previous experiment, we applied the refinement mechanism to the training data, which resulted in a decline in performance. To address this challenge, we conducted two additional experiments, focusing on the test dataset to explore alternative solutions. These experiments correspond to the second scenario outlined in Section 3.3 and **Figure 5B**, and their outcomes are summarized in **Table 3**.

**Table 3:** Results of the refinement mechanism applied as postprocessing.

Method	Kernel size	Predicted masks	Segmentation			Bounding box detection		
			AP_50_	AP_75_	AP_50–95_	AP_50_	AP_75_	AP_50–95_
Baseline	**-**	889 ± 11	91.5 ± 0.4	83.5 ± 0.5	74.5 ± 0.1	91.2 ± 0.3	80.9 ± 1.4	71.4 ± 0.7
GLPF applied to the test dataset	5	869 ± 15	91.2 ± 1.1	82.6 ± 1.3	73.9 ± 0.5	90.4 ± 0.5	79.5 ± 0.4	70.9 ± 0.4
	7	840 ± 38	90.4 ± 1.1	81.6 ± 0.8	72.7 ± 0.4	89.7 ± 1.2	75.8 ± 1.5	72.8 ± 0.4
	9	811 ± 48	89.2 ± 1.0	81.1 ± 1.6	71.4 ± 0.5	88.7 ± 2.0	77.8 ± 1.0	68.5 ± 0.9
HBF applied to the test dataset	5	901 ± 4	92.0 ± 0.8	83.9 ± 0.7	75.1 ± 0.3	91.8 ± 0.5	81.6 ± 0.7	72.0 ± 0.3
	7	909 ± 13	92.3 ± 0.8	83.8±1.2	75.3±0.6	91.9±1.2	82.1±2.6	72.9±2.5
	9	918 ± 2	92.7 ± 0.9	84.5±1.3	75.9±1.1	92.6±1.5	82.8±3.3	73.5±3.8

In this experiment, we employed the refinement mechanism in two different configurations:

#### 4.4.1 Refinement mechanism with GLPF applied to the test dataset

The objective of this experiment was to assess how the presence of instance blurriness in the test data influences the model’s predictions. As revealed by the results in **Table 3**, increasing the kernel size of the GLPF had an adverse effect on both AP and the number of predicted masks. Larger kernel sizes caused the RoIs to become more blurred, resulting in a challenging situation for the model to accurately detect the presence of leaves. The leaves tended to merge with the background, leading to a reduction in overall performance.

#### 4.4.2 Refinement mechanism with HBF applied to the test dataset

In this case, we sought to determine whether applying HBF to the test data, utilizing the refined instances, could enhance the prediction of leaf samples (see **Figure A2 A** in the Appendix for the implemented strategy). As indicated in **Table 3**, by locally applying HBF, the system predicted more leaves, a favorable outcome for downstream processing to obtain phenotypic data. Notably, the AP also improved for both segmentation and bounding box detection, signifying an overall enhancement in performance compared with the baseline.

The results of these experiments demonstrate the advantageous impact of the refinement mechanism, particularly when using HBF. The HBF approach enabled the model to capture more intricate information, resulting in an increased number of correctly predicted leaf instances. While the application of GLPF had a detrimental impact due to increased blurriness, the usage of HBF significantly improved the prediction of leaf instances, contributing to a more effective and precise segmentation.

**Figure 6E** provides an example of a qualitative result, showcasing the visual impact of the strategy on the model’s predictions. This illustration further supports the effectiveness of using the refinement mechanism with HBF in improving leaf instance segmentation in the tomato plant dataset.

### 4.5 Effects of the implemented strategies

#### 4.5.1 Effect of the refined data by HBF

To gain further insights into the contribution and impact of the refinement mechanism, we conducted an in-depth analysis using both GLPF and HBF on the test dataset. First, we applied a GLPF to the test dataset, generating fuzzy instances, and then consecutively applied an HBF to the same areas. For this analysis, we utilized the weights of the model trained with the original augmented images to make predictions on the test data. (See **Figure A2 B** in the Appendix for the implemented strategy).

**Figure 7:**
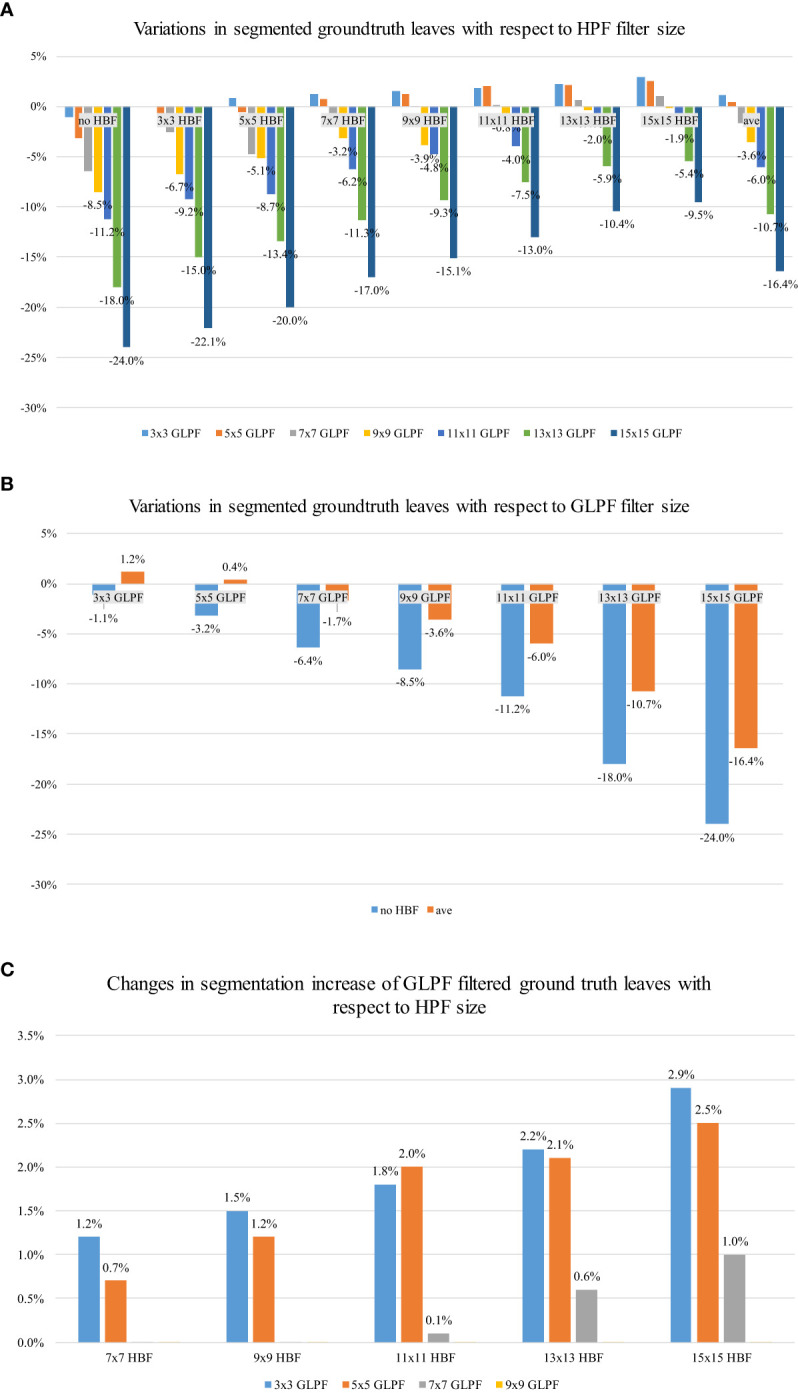
Effects of the implemented refinement strategy on the predicted leaf instances. **(A)** Effect of HBF and GLPF kernel sizes: A kernel size of 15 × 15 positively influenced the model’s performance, resulting in more segmented regions compared with the original test dataset. The “ave” value represents the average change rate across all kernel sizes. **(B)** Effect of GLPF kernel sizes: The level of blurriness had a negative impact on the number of predicted samples. Larger kernel sizes resulted in reduced presence of predicted leaves. **(C)** Improved segmentation of leaves through HBF on GLPF-filtered instances: HBF significantly enhanced the segmentation of leaves, based on the ground-truth labels in the test data, even when blurriness was present in the GLPF-filtered samples.

**Figure 7A** illustrates the changes in the predicted leaf instances based on the size of the HBF core, taking into account the accepted level of blur given by the GLPF. It becomes evident that the model started to benefit from an HBF kernel size greater than 7 × 7 while being constrained by a GLPF kernel size of 3 × 3 or 5 × 5. Furthermore, a trade-off between blurriness and refinement was observed. Larger HBF kernel sizes, such as 15 × 15, exhibited better performance, generating more accurately segmented leaves than those present in the original test data. Additionally, we computed the average change rate (ave) for the GLPF kernel sizes, and it became apparent that the model was generally influenced by more significant levels of blurriness provided by the GLPF.

#### 4.5.2 Effect of the blurred data by GLPF

The effect of the blurred data by the GLPF is depicted in **Figure 7B**, showing the corresponding impact of applying GLPF on the instances of the test data. We used the results obtained with different kernel sizes to measure the changes in predicted leaf instances. Consistent with the findings in Section 4.4, it was observed that the level of blur introduced by the GLPF, based on its kernel size, negatively affected the number of predicted masks. As a result, larger values of kernel size led to a reduction in the presence of predicted leaves.

#### 4.5.3 Effect of the refinement mechanism on the prediction of ground-truth labels

**Figure 7C** complements the aforementioned analysis by showing the performance gain of the predicted instances compared with the ground truth of the test data. The application of HBF substantially improved the predictions regardless of the presence of blur samples. The performance enhancement was found to be dependent on the size of the kernel. Specifically, a 15 × 15 kernel size positively influenced the final results, effectively overcoming the issues caused by GLPF blurring effects.

**Figure 8:**
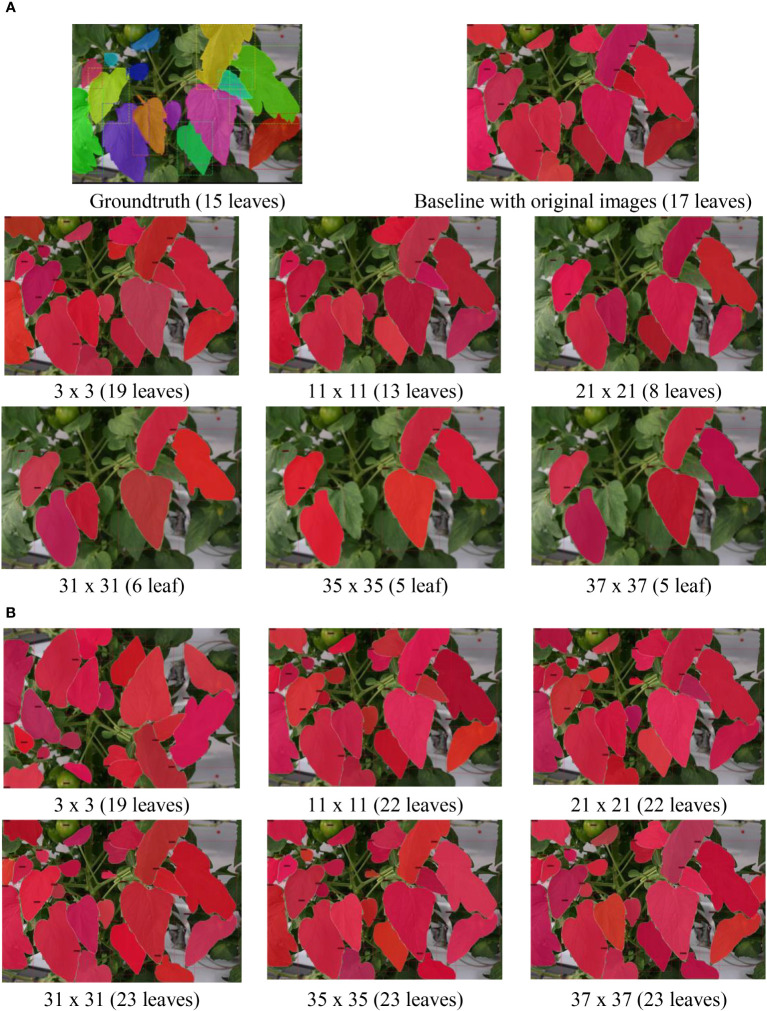
Example results of applying **(A)** GLPF and **(B)** HBF on the test data using an image with multiple leaves. As the GLPF kernel size increased, the prediction performance declined. However, with HBF, the system benefited from larger kernel sizes, resulting in the generation of more accurately segmented leaf instances.

**Figure 9:**
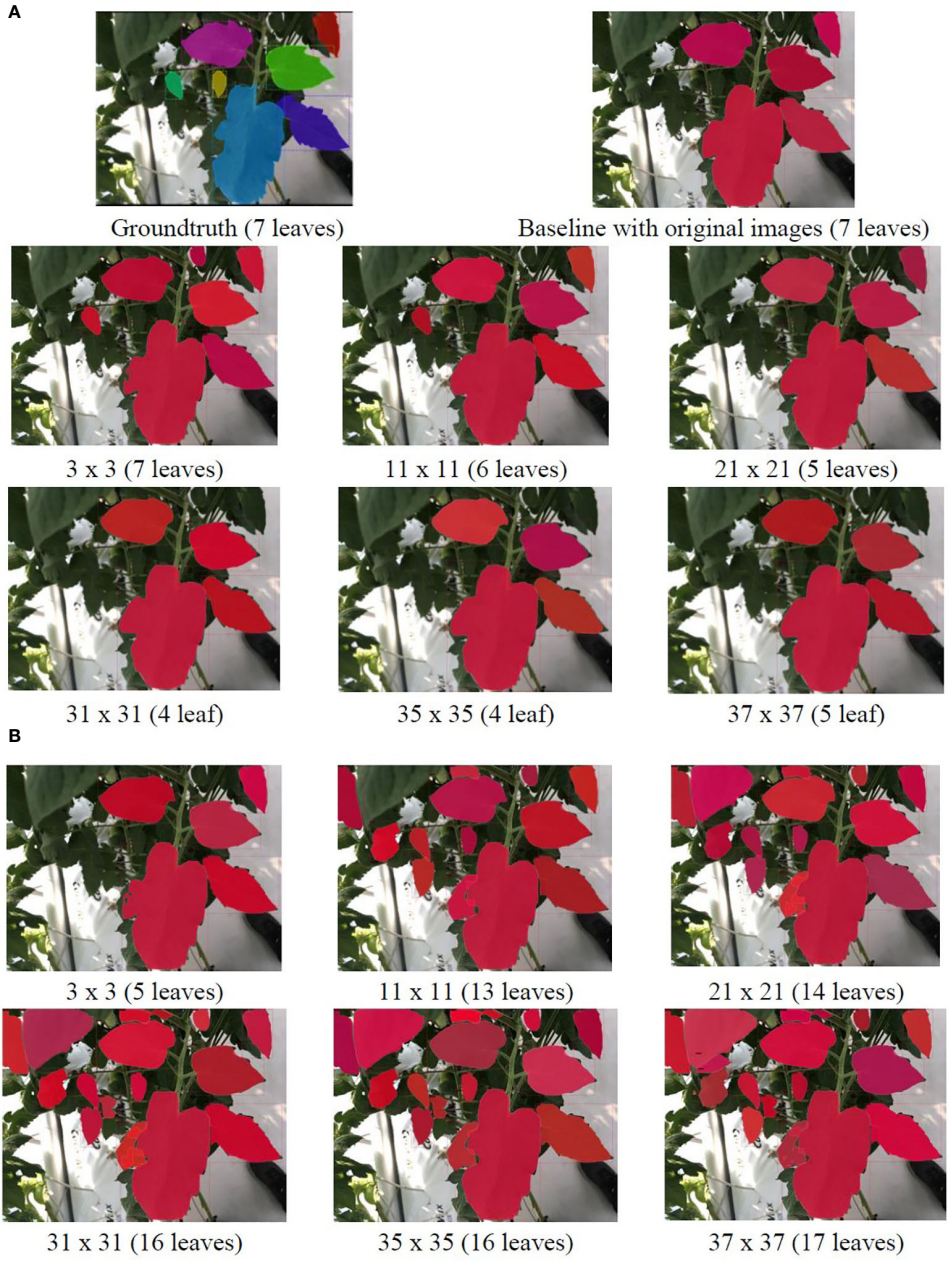
Example results of applying **(A)** GLPF and **(B)** HBF on the test data using an image with few leaves. As the GLPF kernel size increased, the prediction performance declined. However, with HBF, the system benefited from larger kernel sizes, resulting in the generation of more accurately segmented leaf instances.

To visually illustrate the effects of the refinement mechanism on the test data with GLPF and HBF, we present qualitative examples in **Figures 8** and **9**. The figures showcase two cases: one with multiple leaves (**Figure 8**) and the other with few leaves (**Figure 9**). Notably, the use of GLPF and HBF resulted in contrasting performance. While larger kernel sizes of the GLPF negatively impacted the prediction of the ground truth, the larger kernel sizes of the HBF proved beneficial by increasing the number of correctly predicted samples without compromising performance. The HBF effectively enhanced the clarity of RoIs and counteracted the blurring effects of GLPF. Consequently, the model segmented more leaves when the HBF was applied. However, it is important to note that this outcome was highly dependent on the size of the kernel used by the HBF filter.

### 4.6 Comparison with other state-of-the-art architectures

In order to thoroughly assess the effectiveness of our refinement mechanism, we conducted comparative experiments using the HBF on the test data alongside other state-of-the-art methods such as PointRend (Kirillov et al., 2020), Mask Scoring R-CNN (Huang et al., 2019), CARAFE (Wang et al., 2020), Hybrid Task Cascade (HTC) (Wang et al., 2020), Cascade R-CNN (Cai and Vasconcelos, 2018), and Mask R-CNN (He et al., 2017). To ensure fair comparisons, all models were based on the Albumentation transformations method, with (w) and without (w/o) the inclusion of our refinement strategy (Buslaev et al., 2020).

The experimental results, presented in **Table 4**, clearly demonstrate that the proposed refinement strategy significantly improved the performance of all implemented models. Regarding segmentation metrics, Mask R-CNN with the refinement strategy achieved the highest performance with an AP of 92.7% when IoU > 0.5. The HTC model also exhibited comparable capabilities with an AP_50_ score of 92.1% when using our strategy. Notably, the Cascade R-CNN model exhibited the highest improvement of 3.2% after incorporating our refinement mechanism.

**Table 4:** Comparison with other state-of-the-art methods.

Model	Refinement		Segmentation			Bounding box detection		
	w/o	w	AP_50_	AP_75_	AP_50–95_	AP_50_	AP_75_	AP_50–95_
Point Rend	✓		86.9	82.0	76.4	85.8	80.6	74.1
		✓	88.8	85.0	78.4	88.6	82.7	76.1
Mask scoring R-CNN	✓		86.3	79.4	72.4	69.0	86.5	76.9
		✓	87.4	80.5	73.6	87.7	79.7	70.8
CARAFE	✓		89.2	84.4	76.8	88.1	82.3	74.9
		✓	91.2	85.7	78.2	90.0	84.7	76.2
Cascade R-CNN	✓		86.6	82.9	75.9	85.5	80.0	75.1
		✓	89.8	86.1	78.7	89.3	83.0	78.0
Hybrid task cascade	✓		91.8	85.5	77.9	91.4	81.9	75.0
		✓	92.1	86.0	78.4	91.7	83.3	75.7
Mask R-CNN	✓		91.6	83.4	74.5	91.3	81.4	71.6
		✓	92.7	84.5	75.9	92.6	82.8	73.5

In terms of bounding box detection, our improved Mask R-CNN achieved the top score with an AP_50_ of 92.6%. Among the models, Mask Scoring R-CNN displayed the most substantial improvement in performance, with an AP_50_ score of 87.7%, representing an increase of approximately 18.7%. Overall, all models experienced performance gains through the application of our refinement strategy, demonstrating its effectiveness in enhancing leaf instance segmentation in cluttered background conditions.

## 5 Conclusion

This paper introduced an approach for leaf instance segmentation based on deep learning, specifically this research represents a significant step forward in the domain of leaf instance segmentation, offering an innovative and effective approach to tackle the challenges associated with cluttered backgrounds and varying image quality. Through the integration of a local refinement mechanism, we have demonstrated improvements in the accuracy and robustness of leaf instance segmentation. Our proposed refinement mechanism, incorporating Gaussian low-pass and HBF, serves as a key driver behind the effectiveness of our approach. The ability to apply this mechanism either during training or on the test dataset highlights its versatility and adaptability to different scenarios. The refined feature representations within leaf instances enabled the model to better distinguish target leaves, even in the presence of blurriness and cluttered backgrounds. Our qualitative and quantitative experimental results performed on our tomato leaf dataset reinforced the reliability and accuracy of our system in data from real-world greenhouse scenarios. The ability to accurately segment target leaves despite challenging conditions, such as occlusion and overlapping, highlights the potential applications of our approach in plant phenotyping.

## Data availability statement

The raw data supporting the conclusions of this article will be made available by the authors, without undue reservation. For details contact at afuentes@jbnu.ac.kr.

## Author contributions

RM performed the experiments. AF collaborated on the framework design, and data acquisition, and wrote the manuscript. DP and SY advised on the system’s design, analyzed the strategies, and supervised its development. SK, HK, and WL collaborated on the project and its implementation. All authors contributed to the article and approved the submitted version.
